# Assessment of heavy metal release into the soil after mine clearing in Halgurd-Sakran National Park, Kurdistan, Iraq

**DOI:** 10.1007/s11356-018-3597-3

**Published:** 2018-11-14

**Authors:** Rahel Hamad, Heiko Balzter, Kamal Kolo

**Affiliations:** 1 Faculty of Science, Petroleum Geosciences Department, Soran University, Delzyan Campus 44008, Soran, Erbil Iraq; 2 Centre for Landscape and Climate Research (CLCR), Department of Geography, University of Leicester, University Road, Leicester, LE1 7RH UK; 3 National Centre for Earth Observation, University of Leicester, University Road, Leicester, LE1 7RH UK; 4 Soran Research Centre, Soran University, Delzyan Campus, Soran, 44008 Iraq

**Keywords:** Halgurd-Sakran, Heavy metal pollution, Mine clearing, I-geo, EF

## Abstract

The aim of this study was to assess the heavy metal pollution in soils after mine clearance and disposal through controlled explosions in dugout pits during demining operations at two hotspot areas, in the Halgurd-Sakran National Park (HSNP). This investigation was undertaken in order to reveal the concentration level, migration and enrichment in various heavy metals present in polluted soils. Eighteen samples, nine sampling positions at each site, were collected. The current study used inductively coupled plasma-emission spectroscopy (ICP-ES) methodology to determine the concentration levels of Cu, Pb, Zn, Ni, Co, Mn, As and Cr in the soil samples as important toxic contaminants resulting from the demining process. The results show concentration levels of 63.33, 16.22, 116.44, 328, 32.66, 1594.33, 7 and 291.55 ppm in site 1 for Cu, Pb, Zn, Ni, Co, Mn, As and Cr, respectively, while site 2 gave 72.55, 17, 102.55, 296.55, 32, 1851.88, 9.11 and 308.77 ppm. Soil enrichment factor (EF) in sites 1 and 2 of the heavy metals Ni, Cr, Mn, Co and Cu ranged from extremely high enrichment to moderate-high enrichment, respectively. The geo-accumulation (I-geo) index indicated contamination levels that ranged from very strongly to moderately contaminated soil for Ni, Cr, Mn, Co and Cu, respectively. On the other hand, the pollution load index (PLI) showed all values from all samples in both sites were above 1 indicating totally contaminated areas. However, the most polluting heavy metals in the soil at both sites are Ni and Cr with high contamination levels attributed to the controlled mines’ detonations. In conclusion, these mines’ detonations are producing residual heavy metals in the soil that are potentially harmful to the vegetation cover, animals and ultimately humans.

## Introduction

Heavy metals are toxic to soil and to all living organisms (Sardar et al. 2013). The accumulation of heavy metals in soil is a source of concern in agricultural production, because of their negative effects on food safety, crop growth and marketability due to plant poisoning and environmental health of soil organisms (Asati et al. 2016). Mineral constituents, water, air, organic matter and living organisms are the components of soil (Kabata 2010). Therefore, soil represents a complex medium and it is “non-renewable within human time-scales” (Vrščaj et al. 2008). The excess of heavy metals in soils is a significant environmental pollutant that threatens natural ecosystems; thus, it is important to understand their impacts (Kabata 2010). Soil characteristics govern heavy metal mobility through the impact of their chemical speciation and solubility (Liang et al. 2017). Most heavy metals occur naturally in soils as a result of geological processes such as weathering and erosion and the alteration of the geological subsurface materials (Moor et al. 2001). Furthermore, the soils can be a source, basin or reservoir for contaminations that pose a considerable threat to the natural of environment and human health when these heavy metals are released into the environment (Biasioli et al. 2006).

Global development and unplanned agricultural activities have been effecting natural environments and ecosystems (Dantu 2009). Thus, evaluation of soil pollution requires in-depth knowledge of the spatial distribution on contaminants (Barbieri et al. 2017). A polluting metal, which is stable in soil and not absorbed by plants, or insoluble, will have an effect through other processes (Liang et al. 2017) such as inhalation, ingestion and dermal contact or the food chain. Consequently, these heavy metals can be transferred to human bodies, animals and plants (Wang et al. 2012), which are harmful and tend to bio-accumulate in the food chain (Barbieri et al. 2014) that pose risks to humans and ecosystems. The soil structure and properties play an important role in filtering and retaining of toxic elements. Most of the heavy metals are necessary for human health at an appropriate low level, but their excessive amount in soils is toxic to humans, plants and animals (Moldoveanu 2014). The proper assessment of the concentrations of toxic elements in soils is needed in order to lower the high rate of toxic elements in contaminated soils (Moor et al. 2001).

Environmental contamination of soil by minerals directly affects human health (Barbieri et al. 2014). Furthermore, some heavy metals cause serious health problems for humans, such as nickel (Ni), lead (Pb), cadmium (Cd), arsenic (As), chromium (Cr) and mercury (Hg). For instance, nickel is among the elements contributing to the growth of plants, but an excess of nickel accumulation is toxic to humans and other animals. The chances of developing lung, nose and skin cancers can be raised by excessive levels of nickel in soil (Farhadi and Jafari 2016; Farid et al. 2015). Chromium is known to be carcinogenic when the rate in soil is too high and leads to liver and kidney damage (Martin and Griswold 2009). Chromium is also associated with allergic dermatitis in humans (Moldoveanu 2014). Generally, high levels of cobalt (Palit et al. 1994) and manganese (Li et al. 2004) negatively affect plants through a “reduction in plant nutrient, decrease in plant sugar, decrease in chlorophyll concentration, reduction in shoot and root length and slower plant growth”. The human organs such as the nervous system, kidneys, brain and red blood cells are seriously influenced by lead. Kidneys and the liver are affected by cadmium. Arsenic increases the risk of cancer and affects the central nervous system and kidneys. Kidneys would also be affected by mercury, while high amounts of copper can be dangerous to health and it can harm the kidneys and liver and may lead to death. As the water is a good medium for the transport of contaminants in the environment, heavy metals can easily get transported through it (Moldoveanu 2014).

Many soil factors affect the mobility of heavy metals, such as adsorption/desorption, pH, organic matter content, ionic strength, soil texture, pore structure, temperature, the concentration in plants and soil, residual time and translocation (Sherene 2010). Soil pollution by heavy metals is the most resistant to environmental remediation (Sungur et al. 2014); therefore, the reduction of heavy metals in the environment depends on the soil properties (Sherene 2010). There is currently a wide variety and numerous contamination indexes and calculation approaches with a view to evaluate the contamination of soil, such as enrichment factors (EF), pollution load index (PLI) and geo-accumulation index (I-geo). Most of the investigators have drawn the attention by the movement of the heavy metals and their distribution into the soil (Kamani et al. 2015; Zhao et al. 2015).

Many plants are eaten by animals or humans and hence they get into the food chain (Sardar et al. 2013). Thus, heavy metals enter plants, animals and humans through breathing, food and manual handling (Asati et al. 2016), and lastly, these heavy metals have effects on the environment (Sardar et al. 2013).

Many scientists have studied the accumulation of different heavy metals in soils at different locations. Kamani et al. (2015) found the highest average contents of Cu and Zn in industrial soil areas, while Cd in agricultural areas and Pb contents in park areas were the highest. Zhao et al. (2015) studied the transporting of heavy metals in soil to humans through the food chain. They concluded that there would be a negative impact if humans through the food chain consume the heavy metals. Sharma et al. (2009) stated that copper has high mobility in soils and thus less absorbable by soil particles. Finally, Ezeudo (2017) reported that “the greater the metal retardation, the higher the metal retention by the soil, and, thus, the lower the metal mobility”.

Understanding the spatial distribution of land cover types is necessary to assess the effects of heavy metals in soil and identify areas of contamination (Shokr et al. 2016). The interpolation using inverse distance weight (IDW), which is based on the hypothesis that expectations are a linear combination of available data (Xie et al. 2011), can assist the mapping of heavy metal distributions (Shokr et al. 2016).

The effects of landmines not only have impacts on humans but also on the soils’ physically and chemically, through fragmentation of mines and spreading of their toxic materials such as lead, cadmium and nickel into soils following detonations. Mines cause land degradation and pose a major risk to growth as well as the fear they induce in people (Bier 2003; Dobermann et al. 2013; Douglas 2006). Detonating mines by human, animals or through the process of demining destroys the vegetation cover and a fragmented mine affects the bark or root of a plant when exploding (Bajocco et al. 2012; Berhe 2007; Salvati and Bajocco 2011).

The environmental and socio-economic impact of landmines prevents socio-economic expansion and agricultural activities, particularly in rural areas. Douglas (2006) highlighted the destruction of vegetation cover during an explosion. The toxic materials such as cadmium, lead, nickel and mercury spread into the soil, which leads to a high loss of agricultural productivity. Bier (2003) reported the economic impact of landmines through the destruction of livestock, which leads to disrupted markets and food production as a result of the decreasing use of farmland. Dobermann et al. (2013) stated that decreasing farm activity in agriculture and grazing land leads to poverty and scarcity of food and protein. However, a thorough literature review reveals the mobility of heavy metals into soils has caught the attention of scientists.

The objective of this study was to assess the contaminated soils of Halgurd-Sakran National Park in Iraq in terms of heavy metals by determining their concentrations from the samples taken from two different locations after exploding disposal mine processes.

## Materials and methods

### Geographical location

**Fig. 1:**
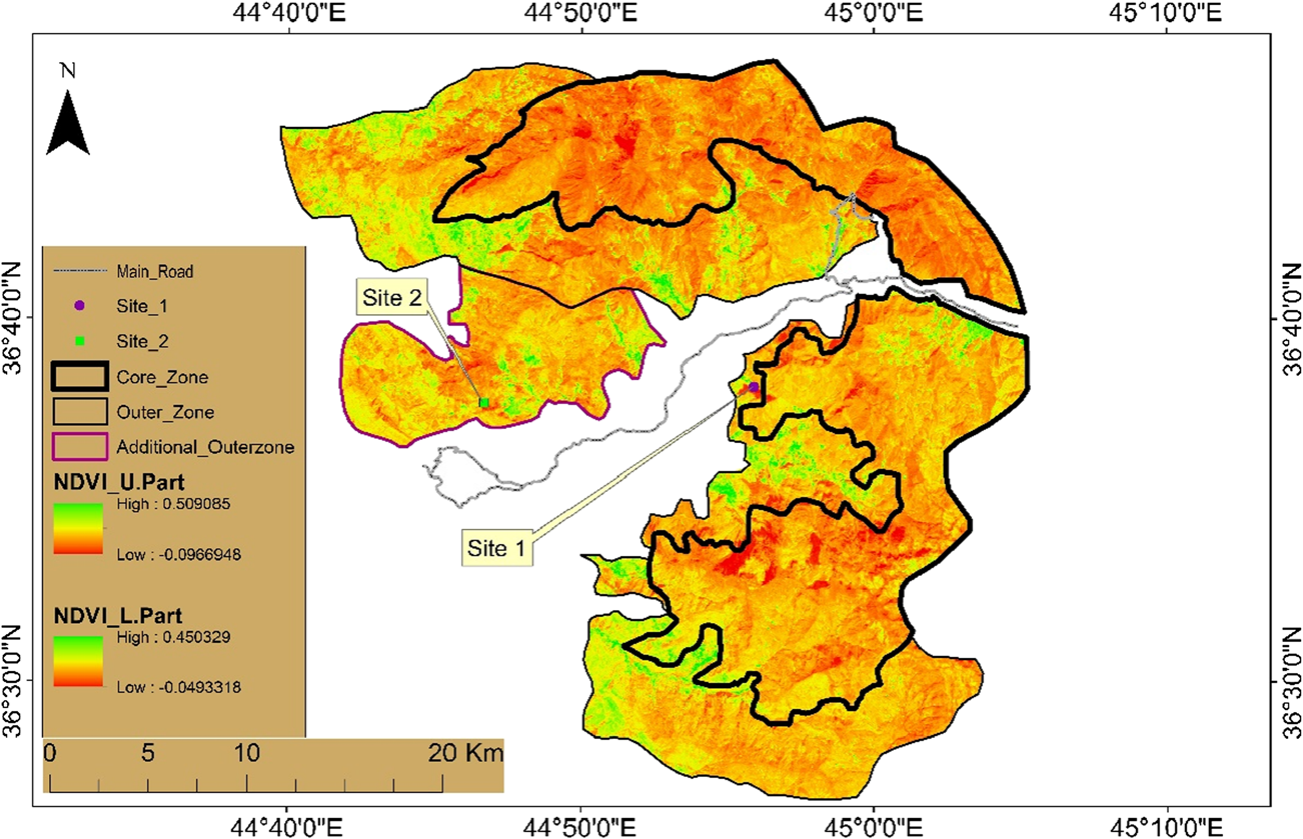
Normalised difference vegetation index (NDVI) and location map of the collected samples at site 1 and site 2, in HSNP

Figure 1 shows the principal geographic subdivisions of Halgurd-Sakran National Park (HSNP), e.g. the Core Zone, Outer and Additional Outer zones (Hamad et al. 2017). Eighteen samples were collected from two detonation sites in HSNP. The sampling was carried out during autumn season under variable ambient temperature of 8 to 25 °C. The soil samples were taken from 0- to 20-cm depths of the soil profile and at 10-m intervals on a grid measuring 20 × 20 m. The samples were sealed and zipped in new plastic polyethylene bags from each sampling site in the same day and sent to the mineral laboratories Bureau Veritas Commodities in Canada. Site 1 is located in the lower part and is a part of the outer core zone of HSNP and is at 15-km distance from site 2, which is located in the upper part and is a part of the additional outer core zone (Fig. 1).

**Fig. 2:**
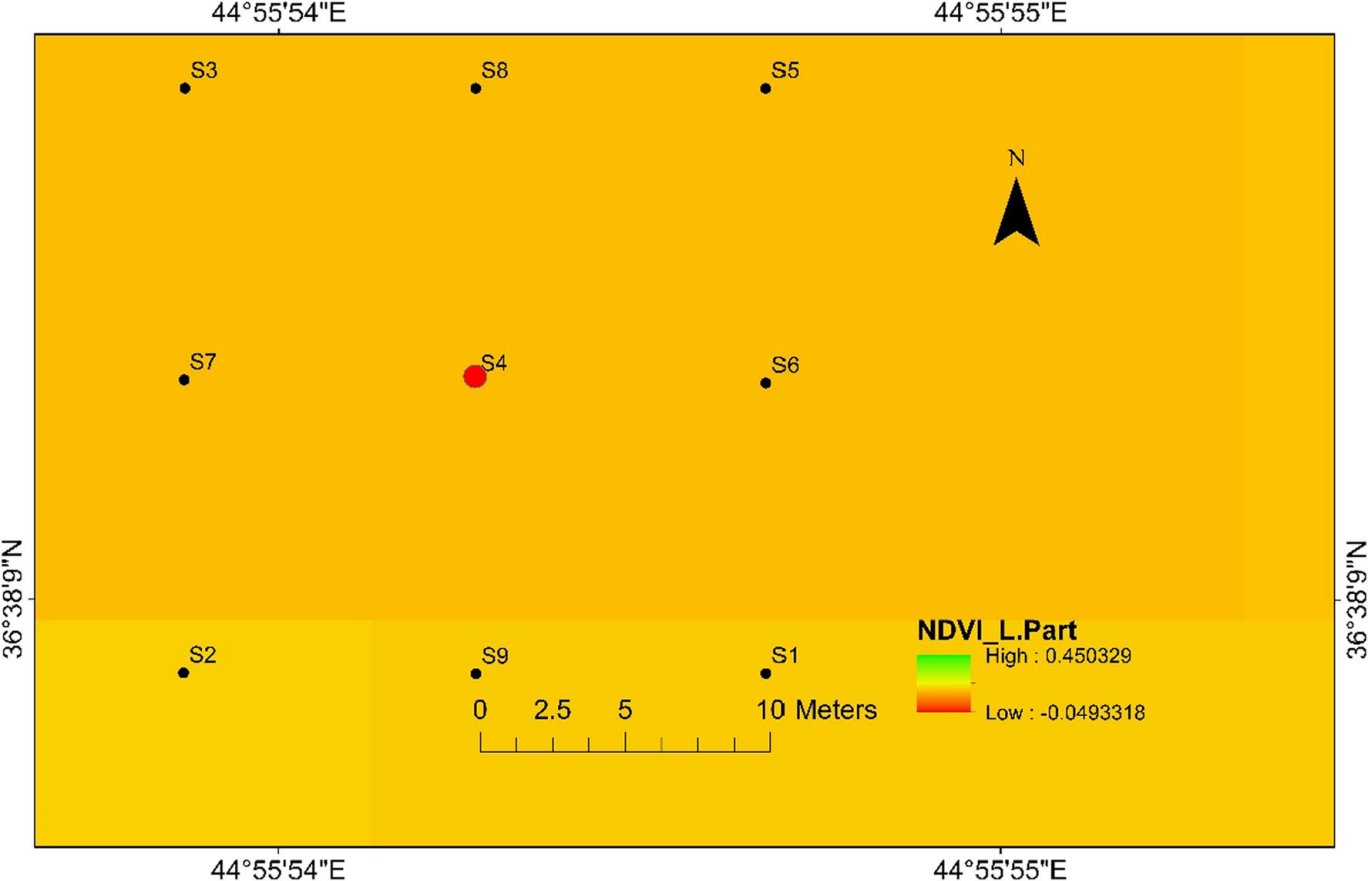
Normalised difference vegetation index (NDVI) map for sampling locations in Halgurd-Sakran Outer Zone. Explosions took place at dugout pit sampling location number 4 at site 1

**Fig. 3:**
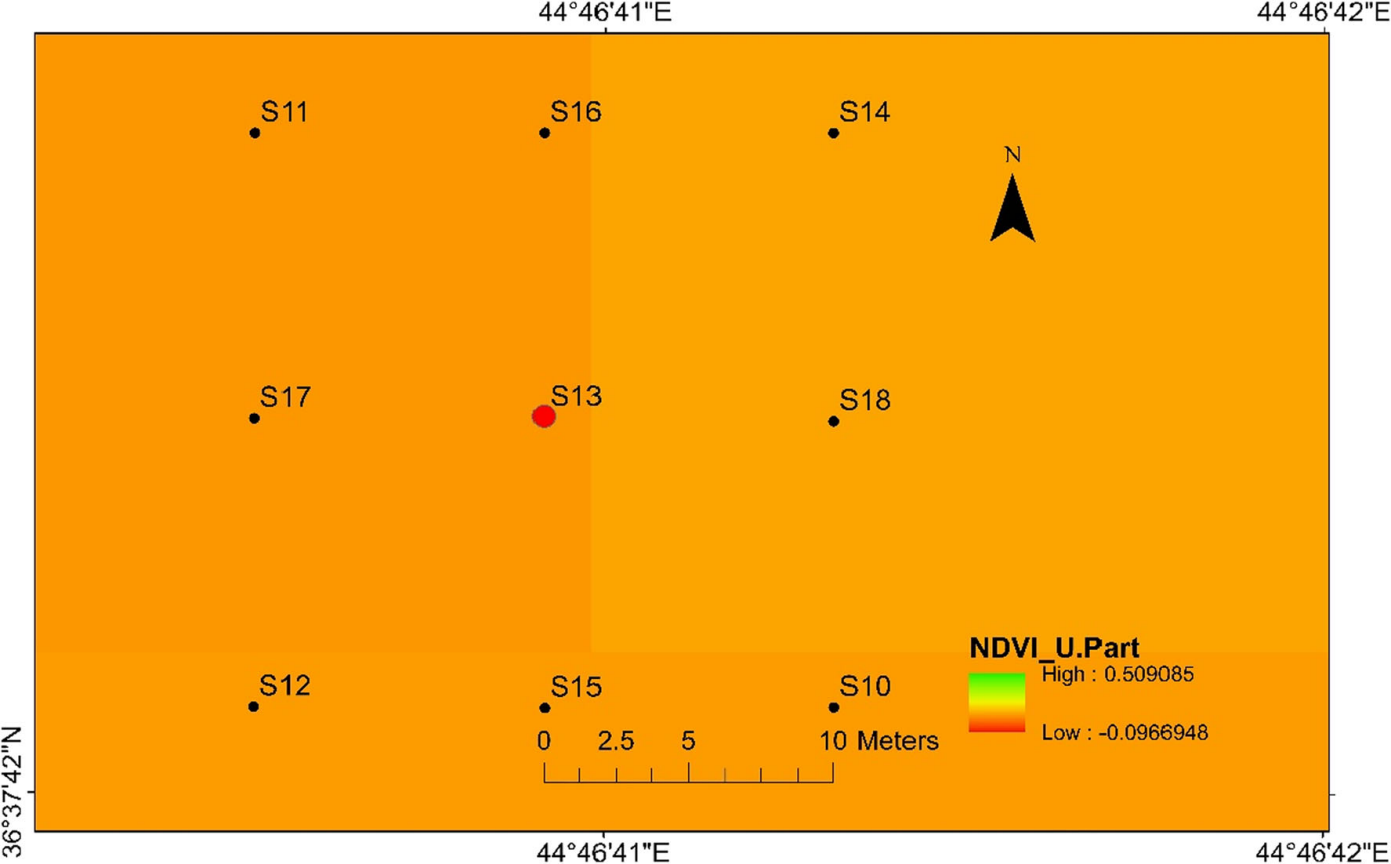
Normalised difference vegetation index (NDVI) map for sampling locations in Halgurd-Sakran Additional Outer Zone. Explosions took place at dugout pit sampling location number 13 at site 2

Moreover, the centre location for site 1 is represented by sample 4 and sample 13 represents the centre of site 2. Specifically, samples 4 from site 1 and 13 from site 2 are places where detonation took place (Figs. 2 and 3). The studied area represents two different crater areas that were used for the explosions away from population settlements.

### Image preparation

Landsat 8 Landsat Data Continuity Mission (LDCM) (path 169/row 035) imagery was acquired on 14 October 2016 with a spatial resolution of 30 m from US Geological Survey (USGS n.d.) Global Visualization Viewer (USGS). The NDVI derived from the satellite data was calculated from the visible and near-infrared light reflected by vegetation. The IDW interpolation of Arc-GIS 10.3 software was used to interpolate the heavy metal concentration in soils over the study area at two sites*.*

### Sample collection and analytical methods

All samples were dried at 60 °C, sieved 100 g, digested in HNO_3_/HCl/H_2_O in the ratio of 1:1:1 and then heat treated. Moreover, prepared sample is digested with a modified *Aqua Regia* solution of equal parts concentrated HCl, HNO_3_ and deionised H_2_O for 1 h in a heating block or hot water bath. Then, samples were analysed using inductively coupled plasma-emission spectrometry (ICP-ES). Analytical procedure and internal references were conducted according to analytical protocol coded OREAS 45Ea and DS11-Beurau Vertasal Mineral Canada (Table 1).

**Table 1:** Limits of detection for the analysis of metals in this survey (ppm)

Element	AQ300 detection (ppm)	Upper limit (ppm)
Ni	1	2000
Mn*	2	10,000
Co	1	2000
Cr*	1	10,000
Cu	1	10,000
Pb	3	10,000
As	2	10,000
Zn	1	10,000

^a^Solubility of some elements will be limited by mineral species present

### Contamination assessment methods

#### Assessment of metal contamination based on enrichment factor

EF is a technique which attempts to differentiate between anthropogenic and naturally occurring sources of heavy metals. The concept of the calculation of potential contamination in soils depends on the concentration of any metal in the topsoil with respect to the reference or background element (Barbieri et al. 2015; Barbieri et al. 2014; Liang et al. 2017). Many researchers used the element aluminium successfully (Allen and Rae 1987; Balls et al. 1997; Loring 1990); therefore, in the present study, aluminium was used as background element in EF formula which defines as follows:

$$ \mathrm{EF}=\frac{\left(\frac{C_x}{C_{Al}}\right) Sample\ }{\kern0.75em \left(\frac{C_x}{C_{Al}}\right) Background} $$

where (C_*x*_/*C*_*Al*_)_*sample*_ represents the ratio of metal and Al concentrations in the sample and (*C*_*x*_/*C*_*Al*_)_*background*_ represents the ratio of element and Al concentrations of the reference.

Category of enrichment factors

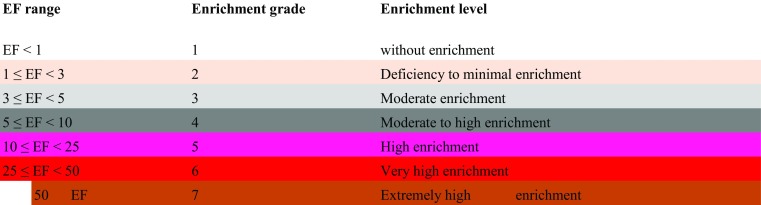



#### Assessment of metal contamination based on PLI

PLI is a quick index used by many authors in order to assess heavy metal contamination in soil and has the formula PLI = $\sqrt[n]{CF1\ x\  CF2\ x\dots . CFn.}$, where *n* is the number of metals and CFs are the contamination factors (Tomlinson et al. 1980). Furthermore, when the PLI value exceeds (1), it indicates polluted soil while a value less than 1 is unpolluted. Soil which is considered “perfect” has a PLI value of 0 (Goher et al. 2014; Moore et al. 2016).

#### Assessment of metal contamination based on geo-accumulation factor

Geo-accumulation was proposed by Muller (1969) and was defined as follows:

$$ \mathrm{I}-\mathrm{geo}=\log 2\ \left[\mathrm{Cn}/1.5\times \mathrm{Bn}\right] $$

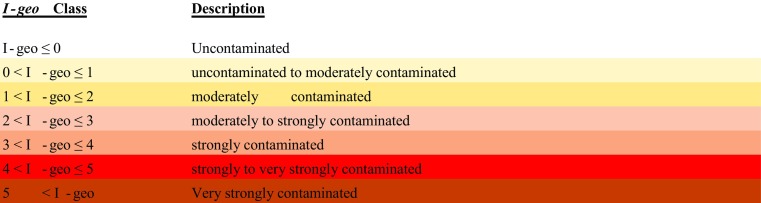

where C*n* is the measured concentration of the element *n* and *Bn* is the geochemical background value element *n* in average crust. The geo-accumulation index has been categorised into seven grades of contaminations ranging from very strongly contaminated to uncontaminated (Barbieri 2016; Muller 1969).

## Results

### Minimum, maximum and mean concentrations of two sites

Table 2 summarises the minimum, maximum, mean concentrations and standard deviation in parts per million for metals Ni, Cr, Pb, Zn, Mn, Co, Cu and As in 18 soil samples collected at two different sites in HSNP. The decreasing trend for averages of heavy metal levels in site 1 was Mn, Ni, Cr, Zn, Cu, Co, Pb and As. Meanwhile, the tendency decreasing for averages of heavy metal levels in site 2 was Mn, Cr, Ni, Zn, Cu, Co, Pb and As (Table 3).

**Table 2:** The concentrations in parts per million and basic statistical parameters (mean, min and max) of heavy metals at site 1 of the investigated soil samples in HSNP

Sample	Cu	Pb	Zn	Ni	Co	Mn	As	Cr
1	54	17	160	182	25	1320	6	140
2	54	18	148	187	26	1316	6	145
3	47	14	134	180	24	1233	6	136
4	56	26	100	832	57	1342	4	462
5	55	14	119	242	28	1425	7	247
6	65	14	106	471	40	1372	8	560
7	87	16	94	317	33	2343	11	353
8	83	15	92	305	33	2217	10	330
9	69	12	95	236	28	1376	5	251
Mean	63.33	16.22	116.44	328	32.66	1549.33	7	291.55
Minimum	47	12	92	180	24	1233	4	136
Maximum	87	26	160	832	57	2343	11	560
SD	13.90	4.08	25.36	210.37	10.416	418.73	2.29	149.43

**Table 3:** The concentrations in parts per million and basic statistical parameters (mean, min and max) of heavy metals at site 2 of the investigated soil samples in HSNP

Sample	Cu	Pb	Zn	Ni	Co	Mn	As	Cr
10	51	18	150	177	25	1372	5	139
11	50	18	124	192	25	1133	7	142
12	78	21	93	306	33	1982	9	335
13	65	17	86	512	42	1285	10	603
14	90	22	96	211	27	2171	10	186
15	88	16	99	264	31	2443	11	249
16	85	17	96	288	34	2355	11	282
17	75	12	93	335	35	2063	10	386
18	71	12	86	384	36	1863	9	457
Mean	72.55	17	102.55	296.55	32	1851.88	9.11	308.77
Minimum	50	12	86	177	25	1133	5	139
Maximum	90	22	150	512	42	2443	11	603
SD	14.85	3.42	21.03	105.65	5.63	478.85	1.96	154.59

### Contamination assessment based on enrichment factors for the heavy metals

The enrichment factor technique was used to evaluate the environmental contamination. Aluminium content in standard earth material was concerned to represent “uncontaminated” reference concentrations, in the present work. The EF of Ni, Cr, Pb, Zn, Mn, Co, Cu and As for site 1 of nine samples were determined and the degree of the heavy metal pollution in the soils was assessed (Table 4).

**Table 4:**
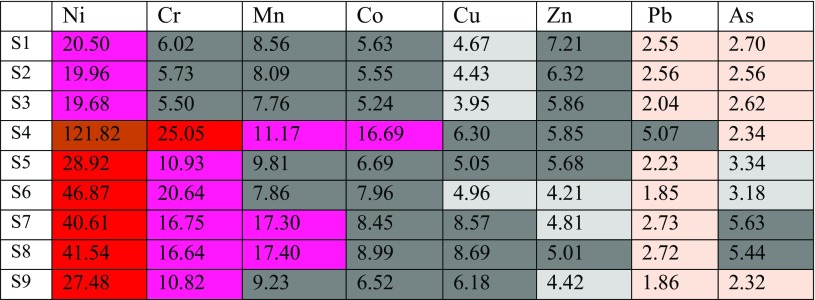
Average enrichment factor of heavy metals in soil samples at site 1 in HSNP

Table 4 shows that Ni had an extremely high enrichment value at site 1 with the highest value of 121.82 ppm in sample 4, followed by sample 13 at site 2 (Table 5) with 50.95 ppm. Once more, Ni had very high enrichment values in five other sampling locations at site 1 ranging from 27.48 to 46.87 ppm as for samples (5–9). Concerning Ni at site 2, six sampling locations fall in a very high enrichment range, samples 12 and 14–18 starting from 30.89 to 49.35 ppm (Table 5), whereas there were three sampling locations at site 1 as for samples (1–3) and two sampling locations samples (10 and 11) at site 2 of Ni which had high enrichment contamination values ranging from 19.68 to 20.50 ppm and 19.38 to 19.56 ppm, respectively (Tables 4 and 5).

**Table 5:**
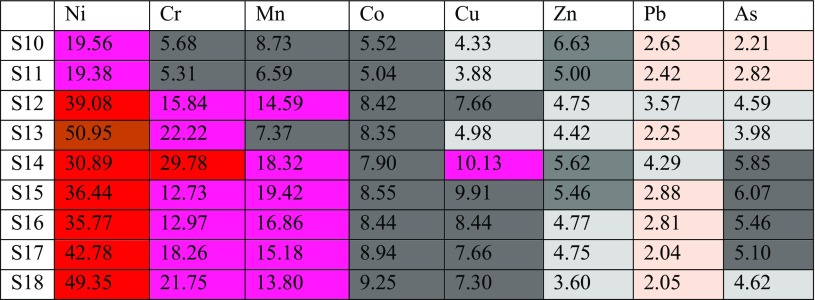
Average enrichment factor of heavy metals in soil samples at site 2 in HSNP

Chrome had a very high enrichment factor in sample 4 at site 1 with a value of 25.05 ppm. At the same time and same site, five samples out of nine fall in the high enrichment class that ranged from 10.82 to 20.64 ppm as for samples 5–9, although the rest of three other samples fall in the moderate to high enrichment range. In terms of site 2, chrome had a very high enrichment factor in sample 14 with a value of 29.78 ppm and high enrichment in samples 12, 13, and 15–18. Sampling locations 10 and 11 fall in the moderate to high enrichment.

The extremely high enrichment factor reflects the major anthropogenic impact on the geochemistry of a certain area, while the very high enrichment has a lesser impact. This is the standard scale for the EF according to specialised literature (Aikpokpodion et al. 2010; Kabata 2010; Manno et al. 2006).

The enrichment factors for manganese at site 2 are higher than at site 1. Moreover, six sampling locations of Mn had a high enrichment factor ranging from 13.80 to 19.42 as for samples (12, 14–18) and the rest of three sampling locations had moderate to high enrichment factor at site 2. Meanwhile, only three sampling locations (4, 7 and 8) out of nine samples fall in the high enrichment factor in site 1 and the rest of the six sampling locations (1–3, 5, 6 and 9) fall in moderate to high enrichment factor.

All sampling locations of cobalt at site 1 fall in the moderate to high enrichment range excluding sample 4 which had the high enrichment factor (Table 4), whereas moderate to high enrichment can be observed at site 2 for all sampling locations of cobalt. Copper and zinc fall in two ranges, which are moderate to high enrichment and moderate enrichment at site 1. Similar to site 1, Cu and Zn fall in the similar ranges excluding sample 14 that had a high enrichment factor of 10.13. A metal that had a very low enrichment was Pb, excluding sample 4 that had moderate enrichment to high enrichment with value of 5.07 at site 1. Samples (12 and 14) at site 2 had moderate enrichment factor and the rest of the samples fall in deficiency to minimal enrichment range of lead. Finally, the As at site 1 embraces three ranges such as moderate to high enrichment samples (7 and 8), moderate samples (5 and 6) and deficiency to minimal enrichment as for the rest of the samples. Similar to site 1, arsenic was present in three ranges at site 2 such as moderate to high enrichment samples 14–17, moderate enrichment samples 12, 13 and 18 and deficiency to minimal enrichment as for samples 10 and 11. Moreover, with regard to mobility, the elements at two studied sites can be arranged for site 1 as Ni, Cr, Mn, Co, Cu, Zn, Pb and As and for site 2 as Ni, Cr, Mn, Cu, Co, Zn, As and Pb.

### Contamination assessment based on pollution load index

PLI was determined for all metals at sites 1 and 2. The results are shown in Tables 6 and 7. PLI is a quick tool to determine the status of the contamination in different locations. In the two sites, the contamination status was observed and the values for all metals were higher than 1, which suggested that the study areas at two sites are totally contaminated. These confirmed that the two sites are facing probable environmental pollution, which was resulting from explosions.

**Table 6:** Pollution load index values in soil samples at site 1 of HSNP

Site 1	S1	S2	S3	S4	S5	S6	S7	S8	S9
PLI	2.58	2.60	2.38	3.83	2.83	2.72	3.63	3.5	2.65

**Table 7:** Pollution load index values in soil samples at site 2 of HSNP

Site 2	S1	S2	S3	S4	S5	S6	S7	S8	S9
PLI	2.51	2.52	3.54	3.79	3.23	3.43	3.20	3.12	3.47

### Contamination assessment based on geo-accumulation index analysis

Three classes of I-geo contamination can be observed for Ni for all sampling locations at site 1 such as strongly to very strongly contaminated class that having the grade of 4.79 in sample 4, strongly contaminated class ranging from 3.01 to 3.97 to in samples 5–8 and moderately to strongly contaminated class for samples 1–3 and 9.

Chrome follows nickel to occupy the second class of I-geo contamination at three different classes, namely moderately to strongly contaminated as in samples (4, 6–8), moderately contaminated as in samples (5 and 9) and uncontaminated to moderately contaminate as for samples (1–3). Mn comes as the third grade of I-geo contamination which seven samples out of nine sampling locations represent moderately contaminated with two locations of moderate to strongly contaminated as for samples (7 and 8). Each of the moderately contaminated and moderately contaminated to uncontaminated classes can be observed for cobalt and copper. Lead and arsenic had less values of I-geo that represent uncontaminated to moderately contaminated and uncontaminated classes.

Table 8 illustrates all aforementioned factors and grades of heavy metals for site 1. In terms of site 2, Ni leads again with its high I-geo value of 4.09 and represents strongly to very strongly contaminated. Strongly contaminated and moderately to strongly contaminated classes can also be observed for Ni in samples 12, 15–18 and samples 10, 11 and 14, respectively. Chrome comes as the second class for I-geo contamination that represents moderately to strongly contaminated samples (14–16) and moderately contaminated area of samples (12, 13, 17 and 18) with two samples (10 and 11) which fell in the uncontaminated class.

**Table 8:**
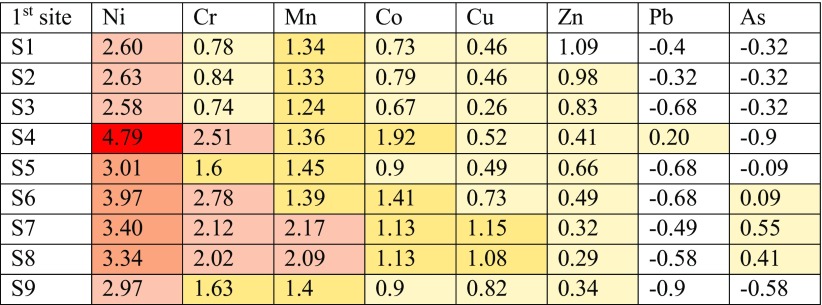
Calculated I-geo index of heavy metals in metals in soil samples at site 1 of HSNP

Three samples (14–16) of Mn fall in the moderately to strongly contaminated class and the rest of samples fall in the moderately contaminated class. Two types of classes can be found for Co and Cu together, which are moderately contaminated and uncontaminated to moderately contaminated. Zinc in all locations falls in the uncontaminated to moderately contaminated of I-geo. All sampling locations of Pb fall in the uncontaminated class excluding sample 11 that fall in uncontaminated to moderately contaminated range. However, six sample locations of arsenic fall in the uncontaminated to moderately contaminated range and the rest three sampling locations (10, 11 and 16) in uncontaminated grade, as presented in Table 9.

**Table 9:**
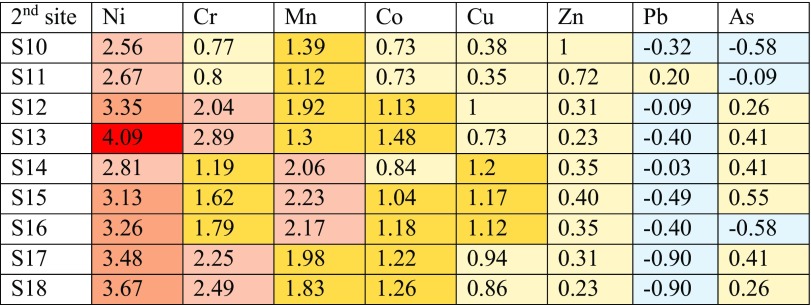
Calculated I-geo index of heavy metals in soil samples at site 2 of HSNP

### Assessment and mapping of heavy metal in soils in HSNP

In this work, the spatial distributions and the concentration levels in soils of eight heavy metals, namely Ni, Co, Cr, Zn, Mn, As, Cu and Pb at two sites, were investigated.

#### Nickel

**Fig. 4:**
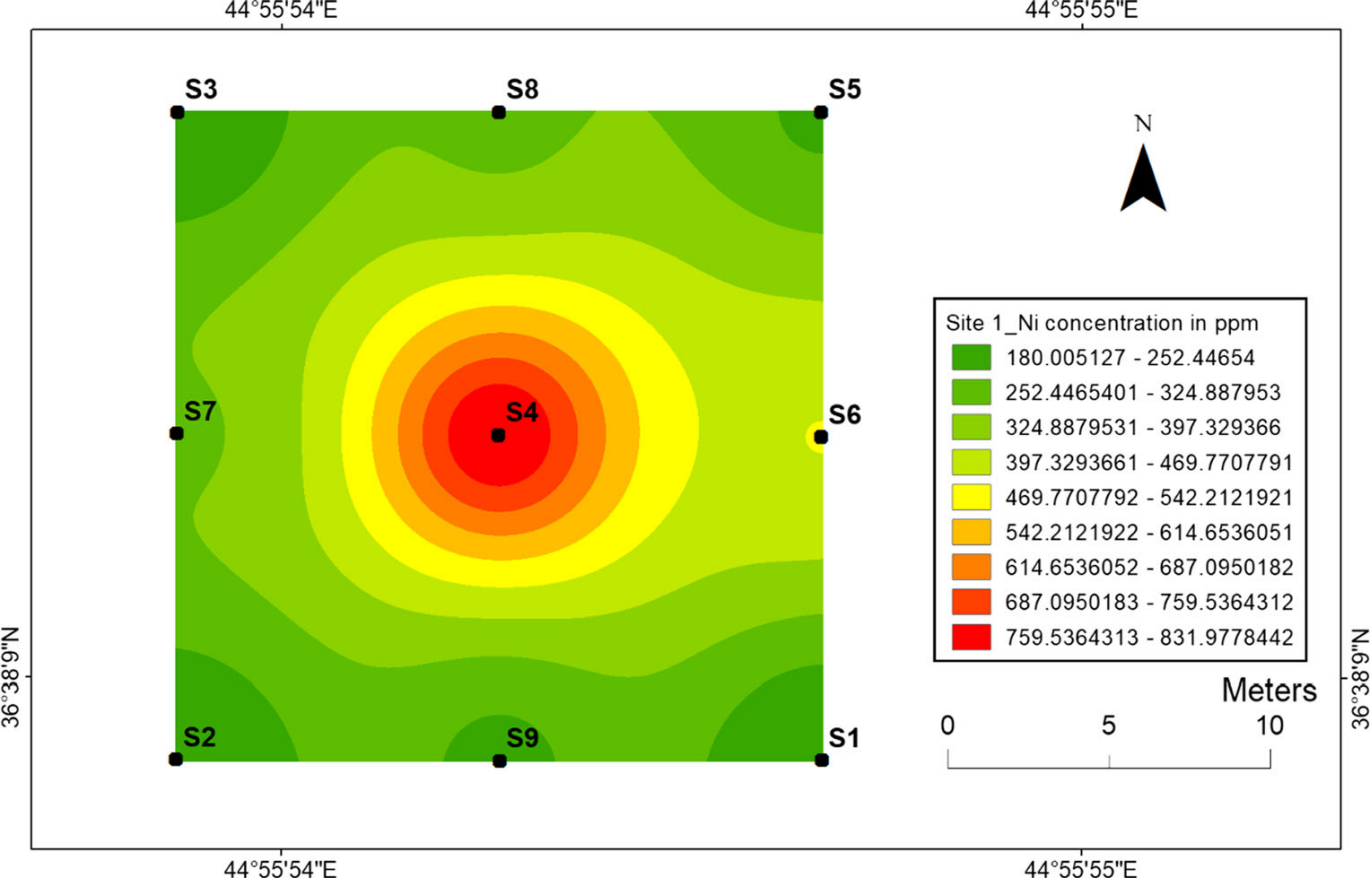
Spatial distribution of nickel in the surface soils at site 1 in HSNP

**Fig. 5:**
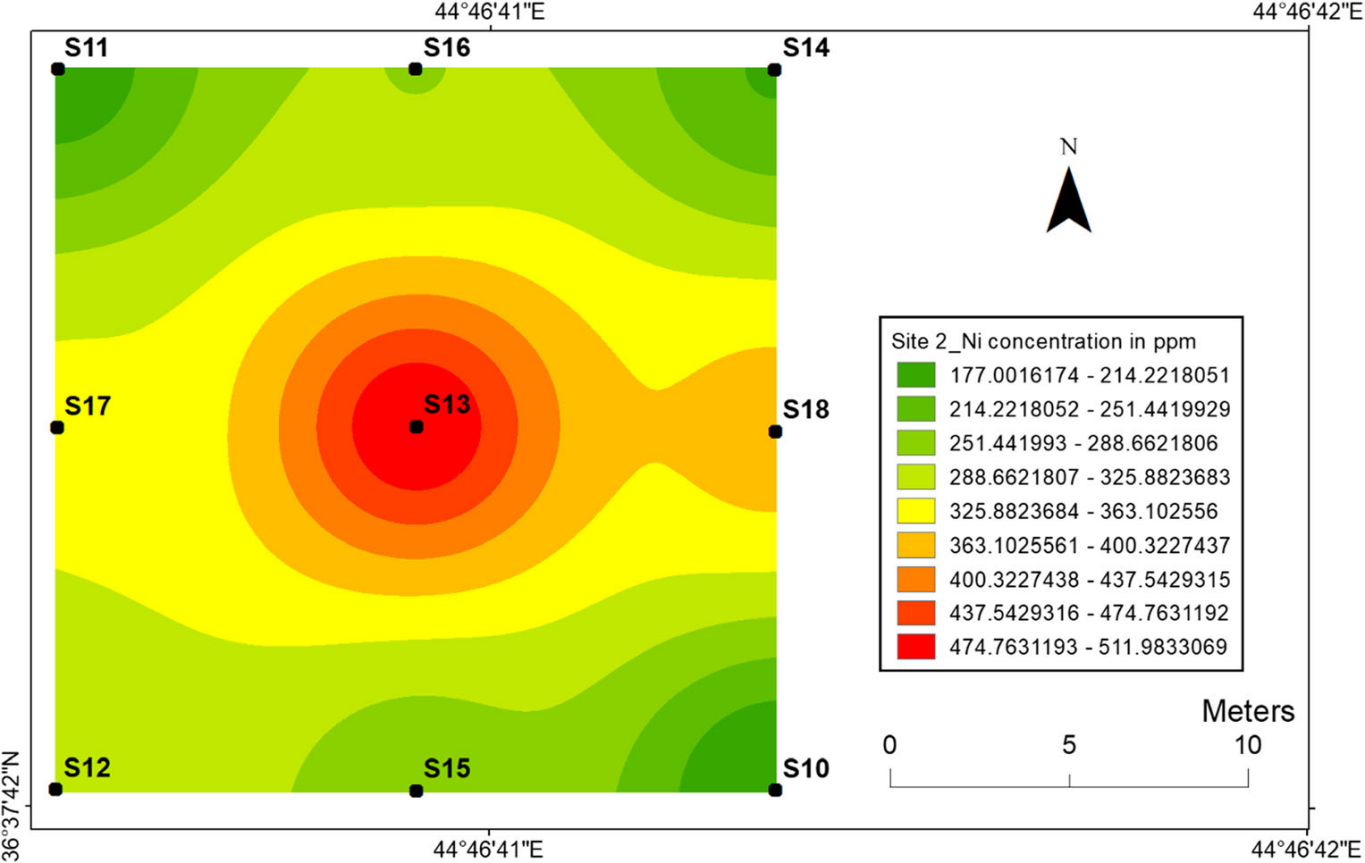
Spatial distribution of nickel in the surface soils at site 2 in HSNP

The highest nickel concentration (832 ppm) in the top soil samples has been found at location 4 at site 1 (Fig. 4) and at location 13 with value of 512 ppm at site 2 (Fig. 5). Meanwhile, the lowest nickel concentration (180 ppm) has been found at location 3 at site 1 and 177 ppm has been found at location 10 for site 2. The high Ni values in the centrals are associated with the high concentrations of heavy metals released into the soil during the explosions of mines and are also associated with low mobility of Ni. Therefore, the topsoil maps for Ni at two sites are very similar with concerning their distribution in the soil.

#### Cobalt

**Fig. 6:**
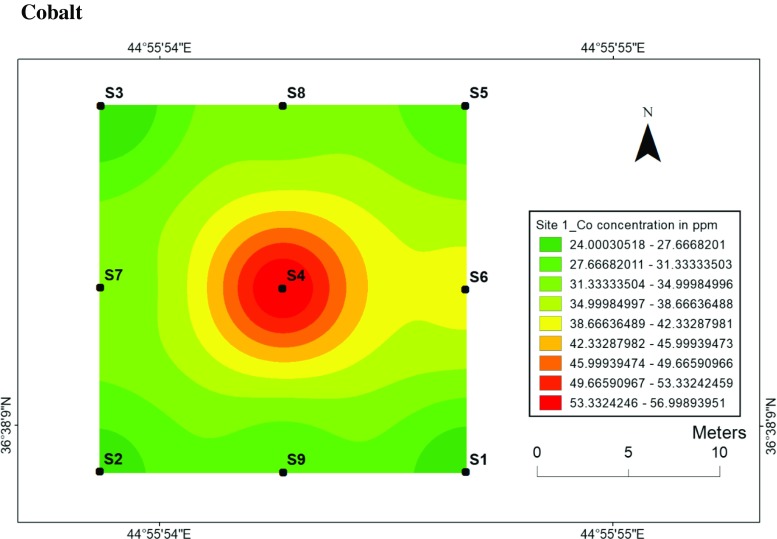
Spatial distribution of cobalt in the surface soils at site 1 in HSNP

**Fig. 7:**
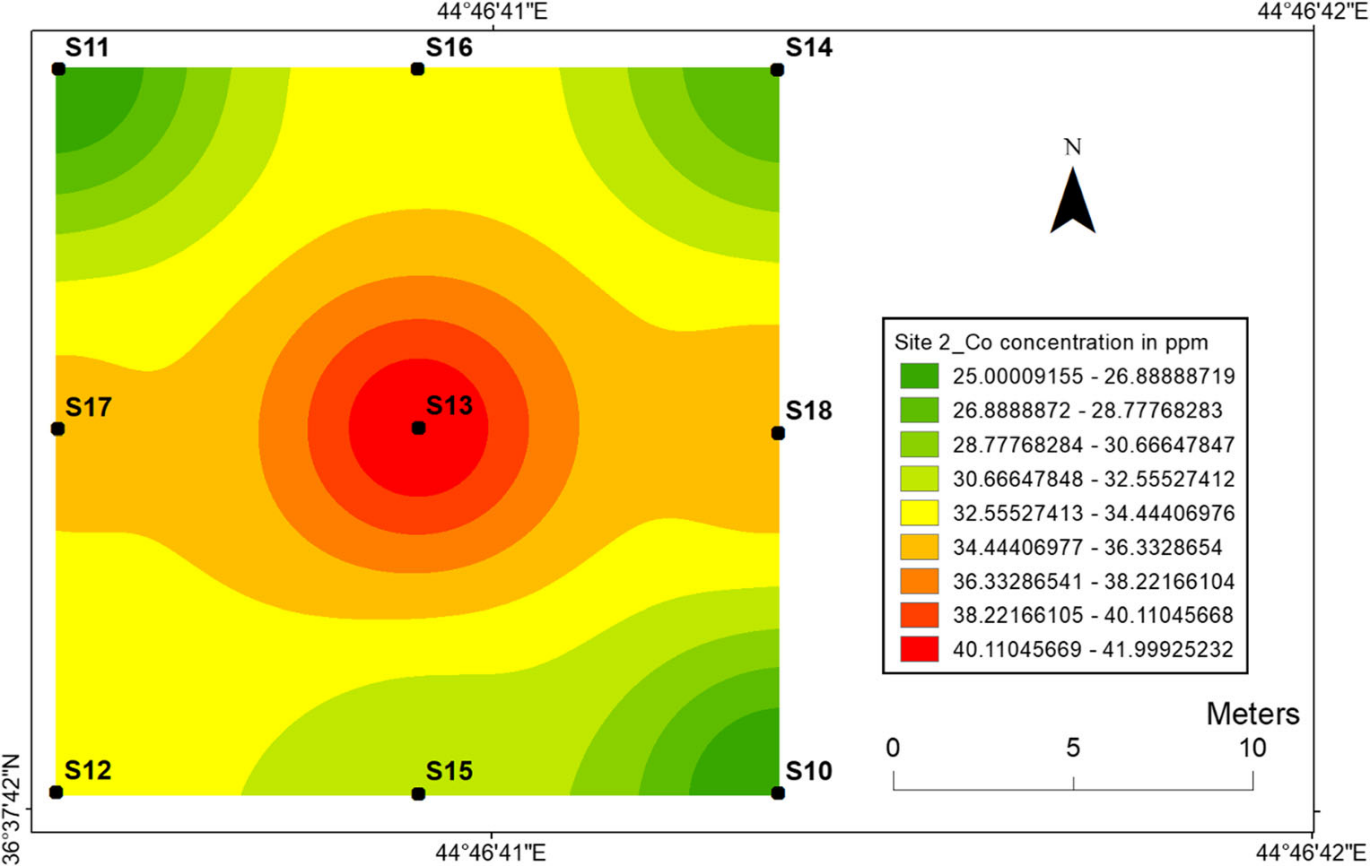
Spatial distribution of cobalt in the surface soils at site 2 in HSNP

Similar to the nickel, the topsoil maps of cobalt at two sites have almost the same distribution as the Co concentrations are higher in centrals at sites 1 and 2. The concentration of Co at site 1 was 57 ppm at location 4 with the lowest value of 24 ppm at sampling location 3 (Fig. 6), while the highest value of Co for site 2 was 42 ppm at the central location with the lowest value of 25 ppm at sampling location 11 (Fig. 7). The significance of the higher concentrations of Co at sites 1 and 2 can be also explained by the release of Co from the detonating mines. The immobility of Ni and Co at two sites can be attributed to the increase of pH due to the precipitation of hydroxides and the formation of insoluble organic complexes.

#### Chromium

**Fig. 8:**
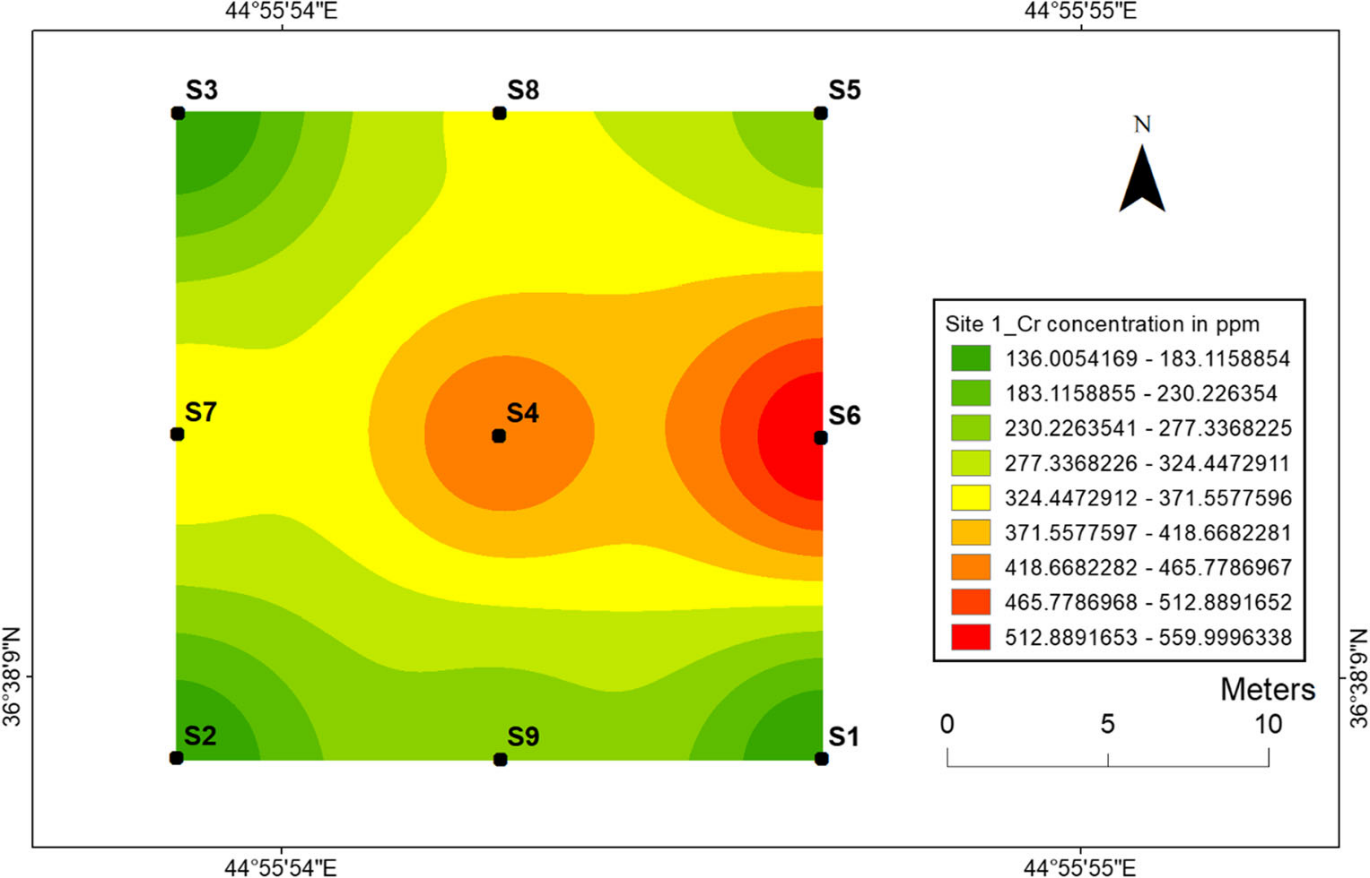
Spatial distribution of chromium in the surface soils at site 1 in HSNP

**Fig. 9:**
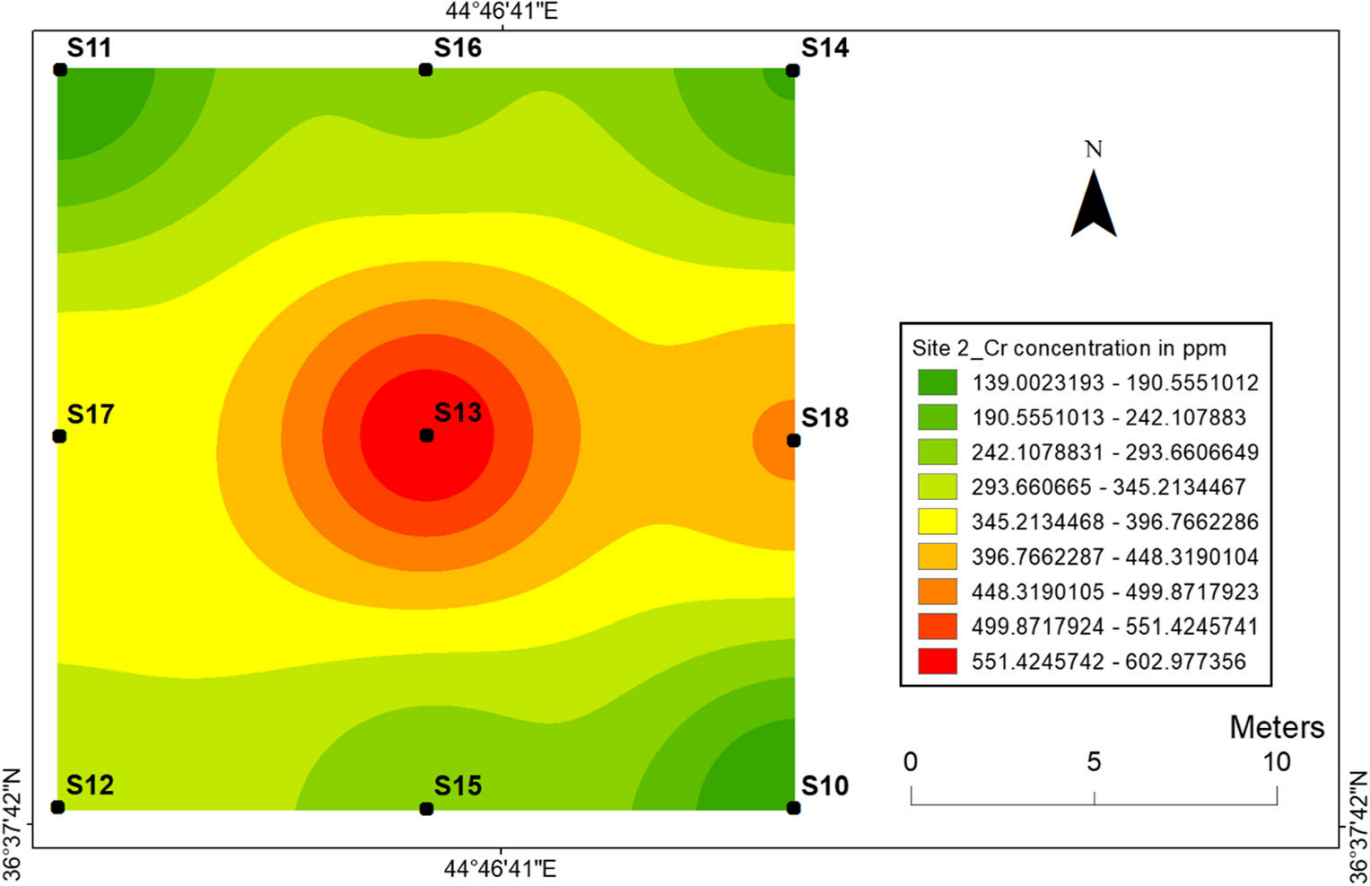
Spatial distribution of chromium in the surface soils at site 2 in HSNP

Concerning chromium, with regard to the explosions conducted at centrals, the distributions of Cr at two sites are not similar such as Ni and Co. Maximum concentration of chromium was obtained at the central point of site 2 (603 ppm) (Fig. 9). However, the highest value at site 1 was observed at location 6 (560 ppm) not at central location 4 (Fig. 8). Chromium transportation in the soil showed that its mobility was highly immobile at site 2, while there was a slow translocation of Cr at site 1. This can be linked to the factors such as pH, sorption of Cr and time dependence between two sites (Fig. 9).

#### Zinc

**Fig. 10:**
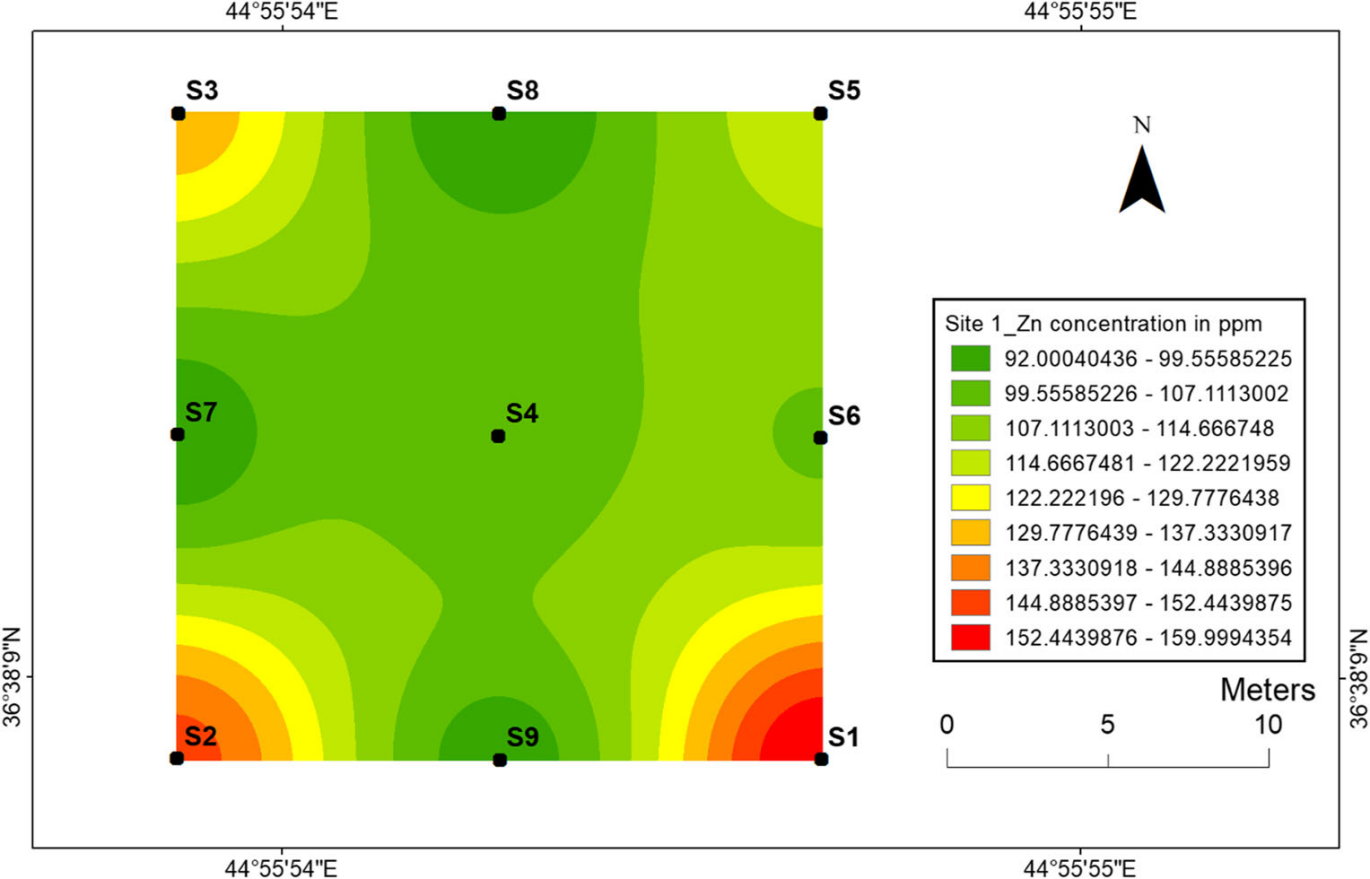
Spatial distribution of zinc in the surface soils at site 1 in HSNP

**Fig. 11:**
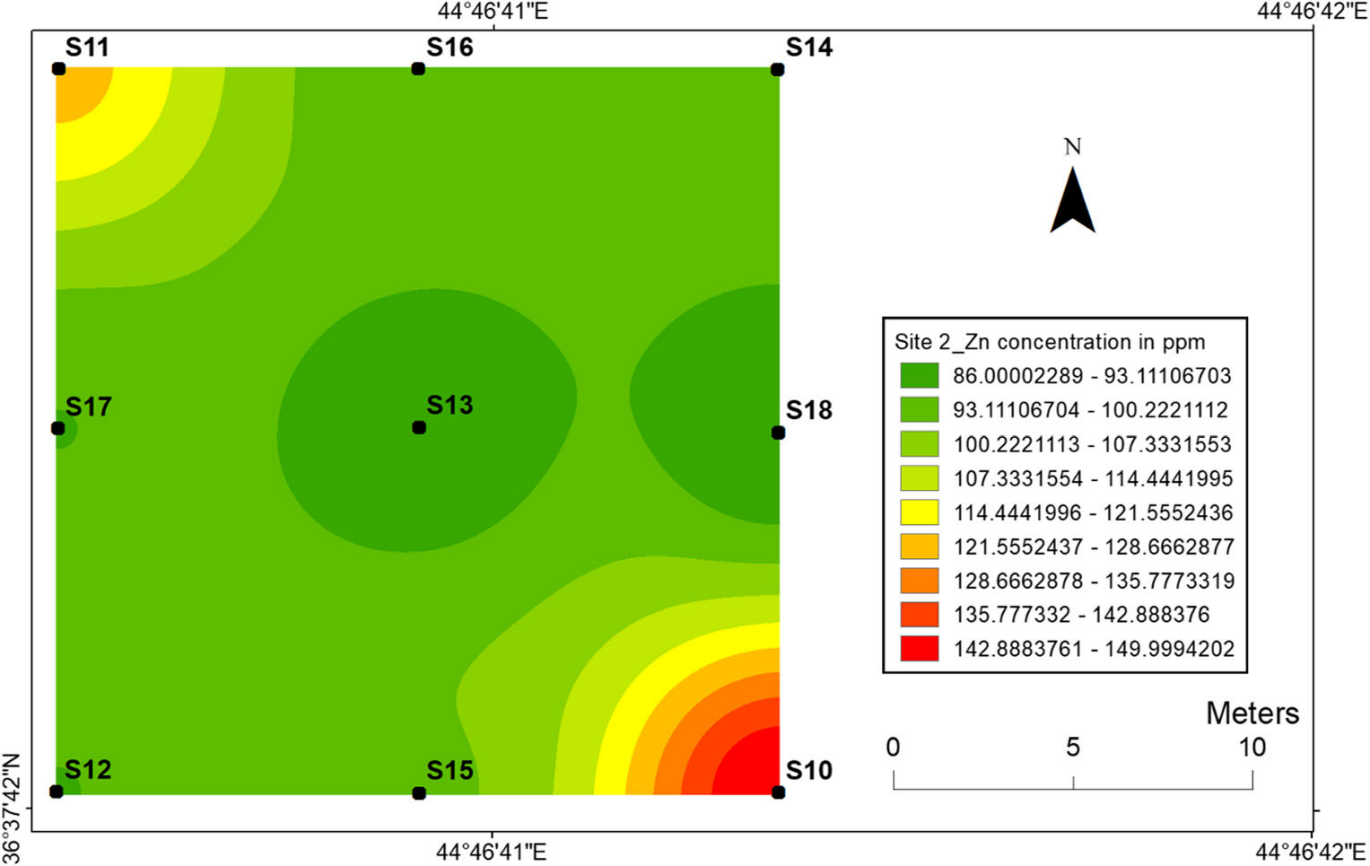
Spatial distribution of zinc in the surface soils at site 2 in HSNP

The concentrations and geographical distributions of zinc in soil for two sites are given in (Figs. 10 and 11). Zinc concentrations ranged from 92 to 160 ppm at site 1 and from 86 to 150 ppm of site 2. Furthermore, Fig. 10 displays the highest zinc concentration that has been found at location 1, while Fig. 11 shows the highest zinc concentration at location 10 of site 2. The lowest zinc concentration 92 ppm was found at location 8 for site 1, whereas two location samples 13 and 18 had the lowest values of Zn. The results show that zinc mobility in the soil was so great and there was greater accumulation of Zn around dugout pits. However, almost the distributions of the Zn were similar at two sites. Thus, very little zinc was absorbed by the soil from soil surfaces, which can be related to the retardation factor.

#### Manganese

**Fig. 12:**
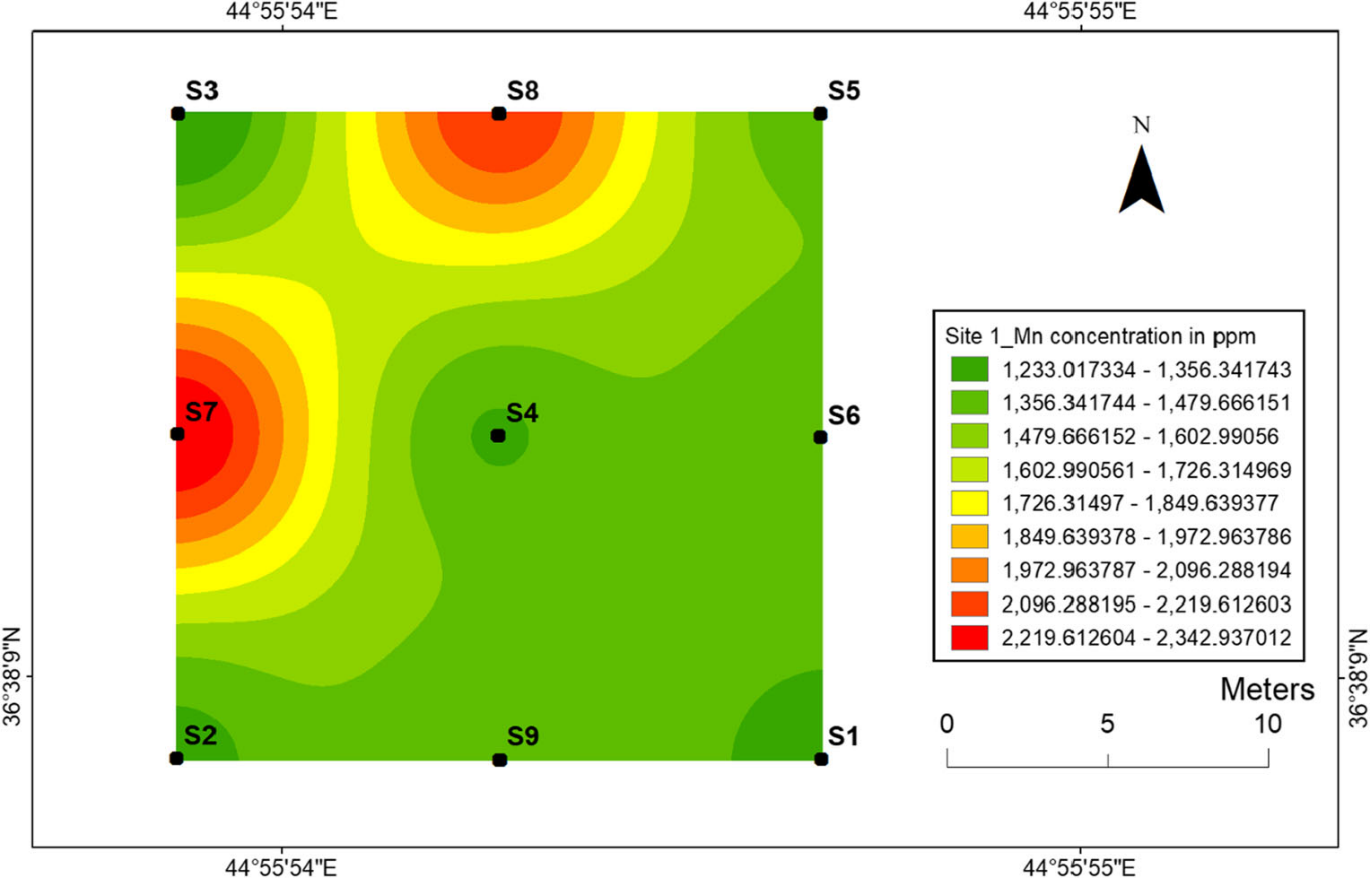
Spatial distribution of manganese in the surface soils at site 1 in HSNP

**Fig. 13:**
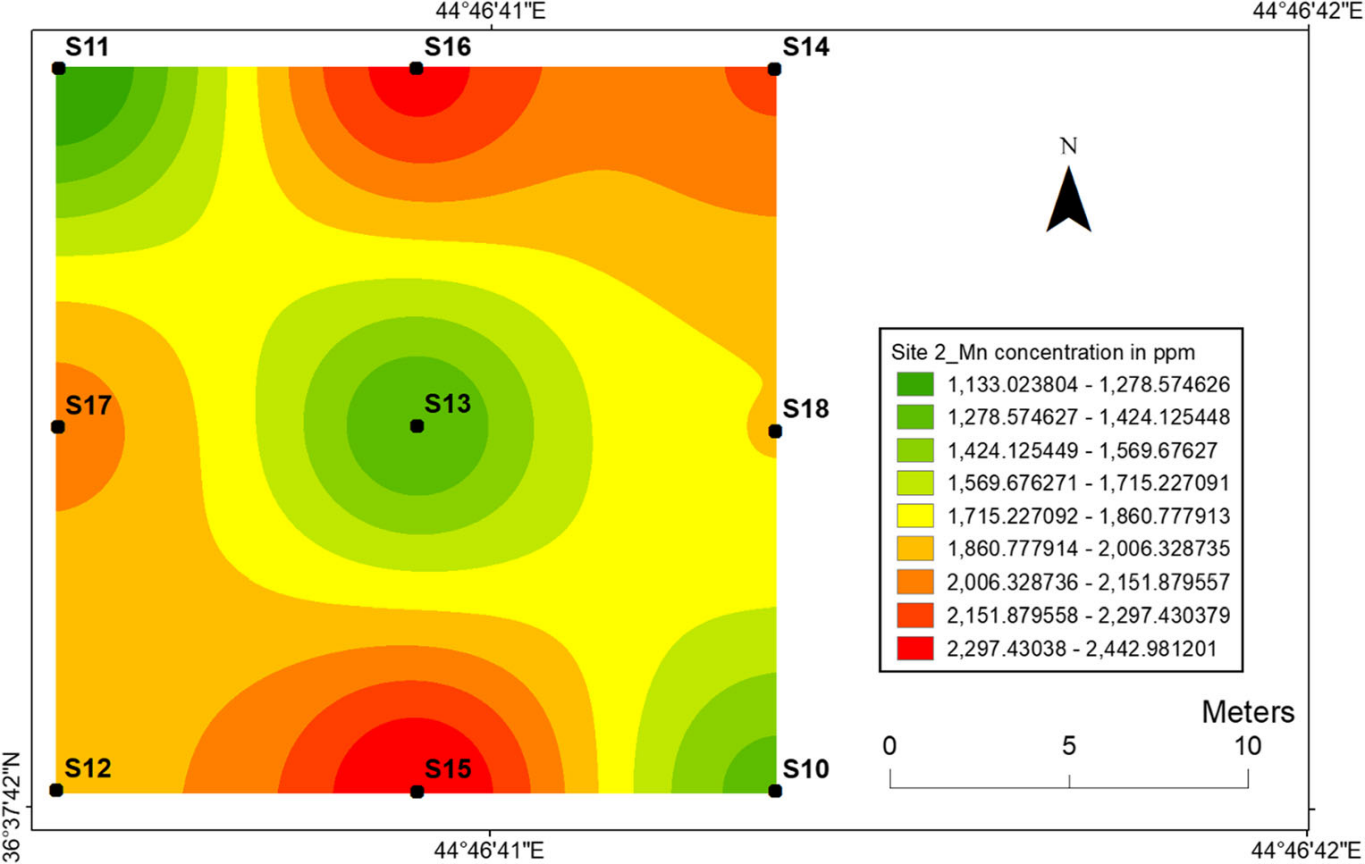
Spatial distribution of manganese in the surface soils at site 2 in HSNP

The highest value of manganese concentration was determined at sampling location 7 for site 1 and at sampling location 15 for site 2 (Figs. 12 and 13), respectively. The presence of soil organic matter and its fraction significantly increased the mobility of manganese ions in soil at two sites. Manganese is a real threat to the human health through high amount exposure in human’s body. For example, the excess of Mn in the brain causes neurotoxicity. It is also toxic to the environment which decreases the fitness of the organism and causes root browning of the plants which indicates the presence of oxidation (Ye et al. 2017).

#### Arsenic

**Fig. 14:**
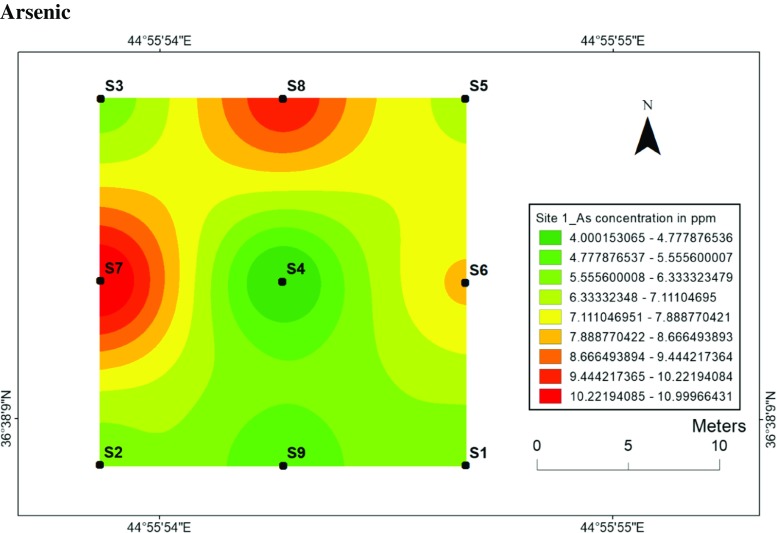
Spatial distribution of arsenic in the surface soils at site 1 in HSNP

**Fig. 15:**
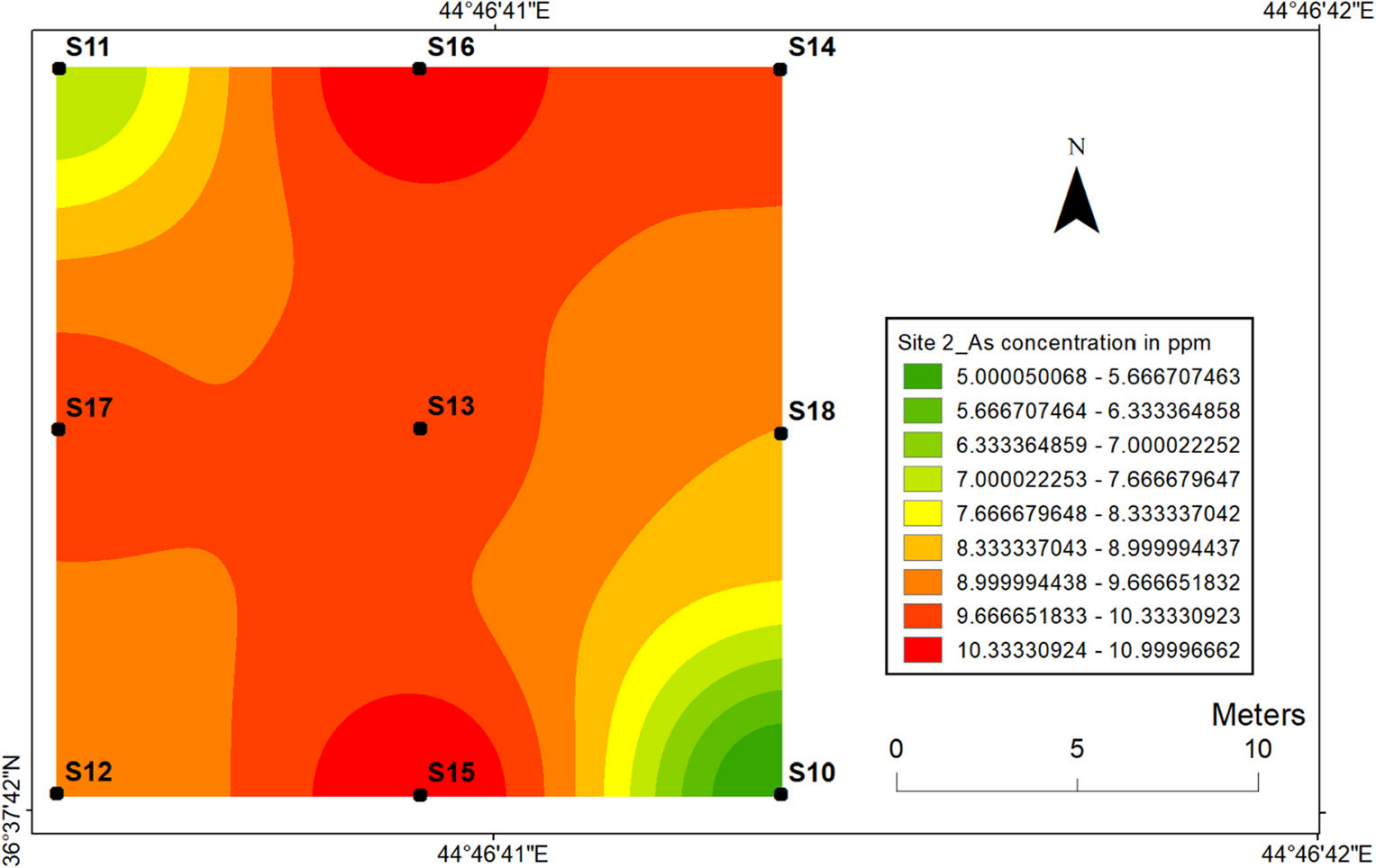
Spatial distribution of arsenic in the surface soils at site 2 in HSNP

Concentrations of arsenic ranged from 4 to 11 ppm at site 1 and 5 to 11 ppm at site 2 (Figs. 14 and 15), respectively. The distribution of arsenic differs between two sites. For instance, the lowest concentration of arsenic was observed at central (location 4), while at site 2, the centre or dugout pits had almost high concentration of arsenic. Furthermore, the mobility of arsenic in site 1 is higher than site 2; therefore, much higher concentrations have been measured in and surrounded sampling locations at site 2. This can be highly dependent on the solubility and the size of particles of arsenic in soil for site 1.

#### Copper

**Fig. 16:**
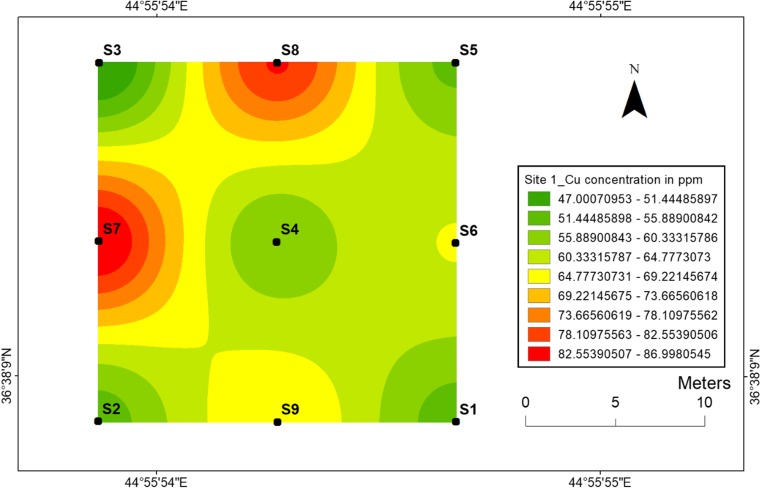
Spatial distribution of copper in the surface soils at site 1 in HSNP

**Fig. 17:**
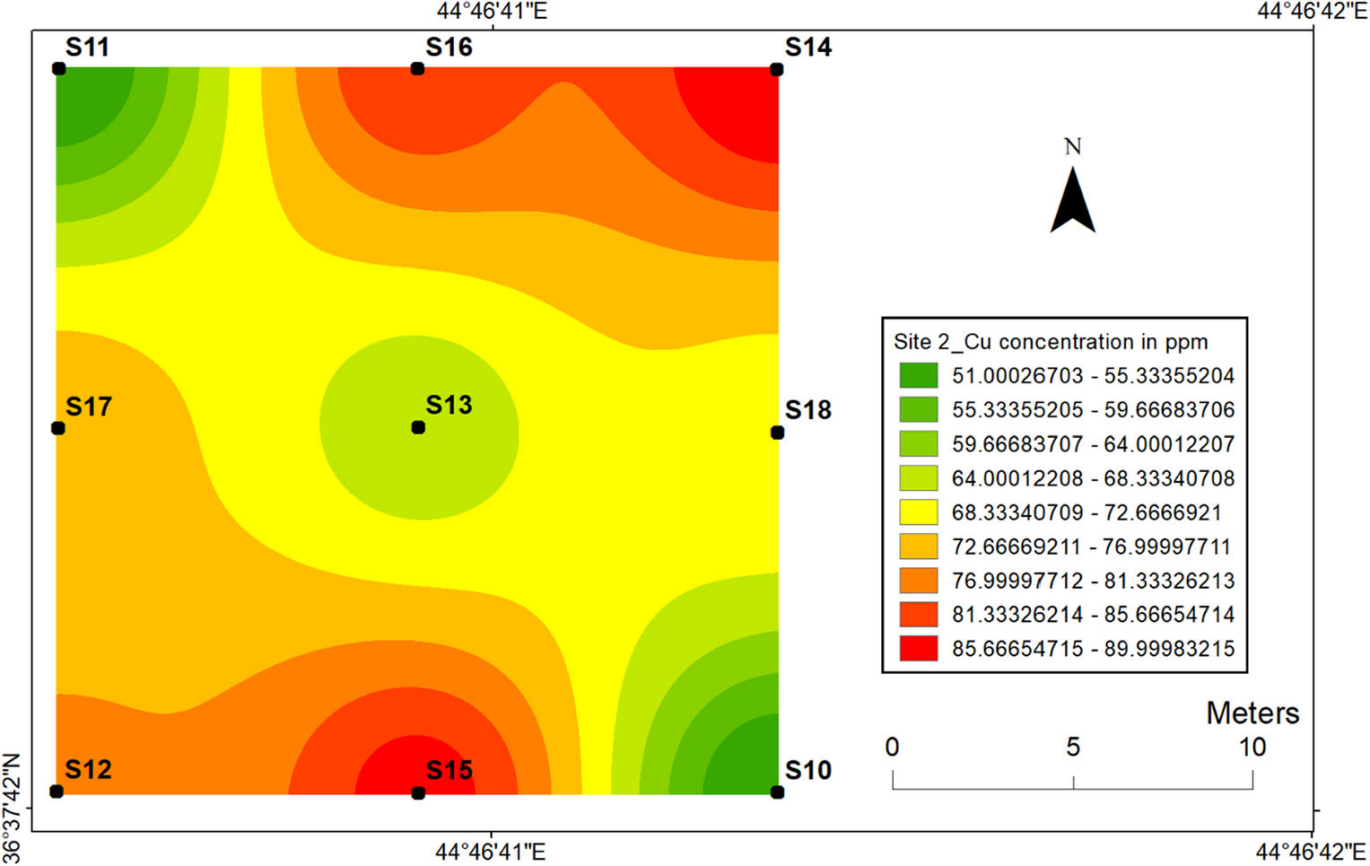
Spatial distribution of copper in the surface soils at site 2 in HSNP

The lowest concentration (47 ppm) of Cu in site 1 was observed in the sampling location 3 with the higher value of 87 ppm at the sampling location 7 (Fig. 16), whereas the highest concentration (90 ppm) of Cu at site 2 was observed at sampling location 14 and the lowest (50 ppm) value was observed at sampling location 11 (Fig. 17). Sonmez et al. (2006) reported a decline in the growth rate of tomato plants after an excessive amount of Cu to a nutrient medium.

#### Lead

**Fig. 18:**
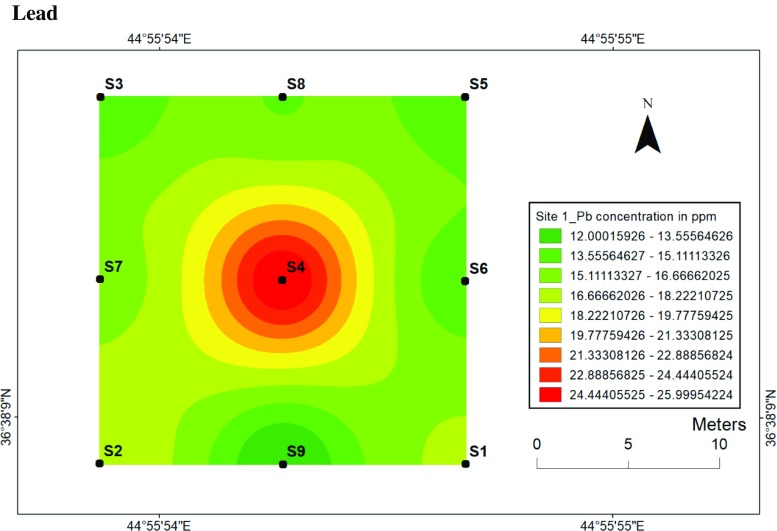
Spatial distribution of lead in the surface soils at site 1 in HSNP

**Fig. 19:**
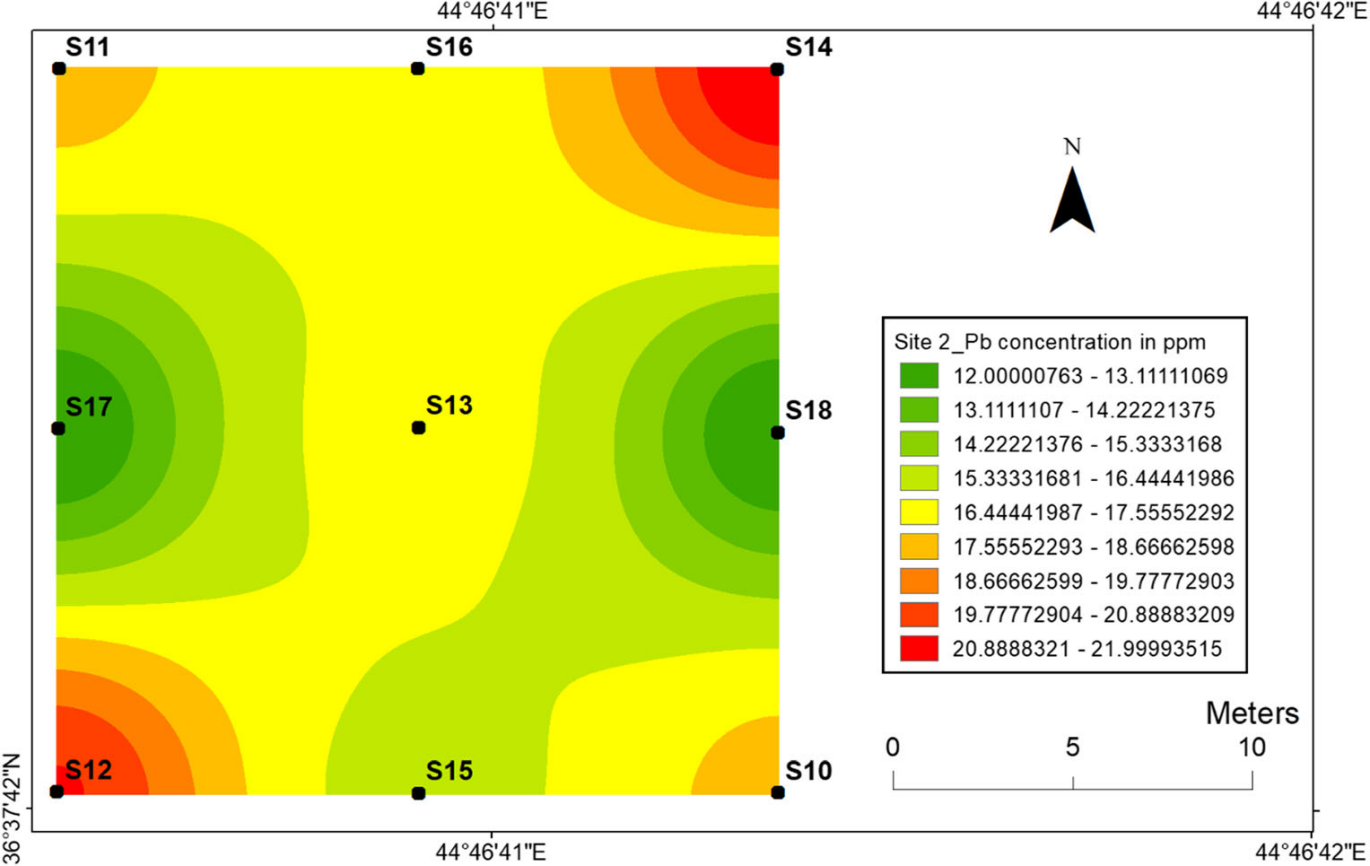
Spatial distribution of lead in the surface soils at site 2 in HSNP

Lead concentrations ranged from 12 to 26 ppm at site 1 (Fig. 18) and from 12 to 22 ppm at site 2 (Fig. 19). Lead concentrations at site 1 accumulated at the centre; thus, the mobility of Pb is too slow compared to site 2 as the accumulated Pb can be observed far away from the centre. Moreover, the degrees of soil development and leaching processing of Pb at two sites are not the same, which affects mobility in soils. This can be explained by many factors such as pH, surface complex formation, ionic exchange, temperature, grain size and adsorption processes.

**Fig. 20:**
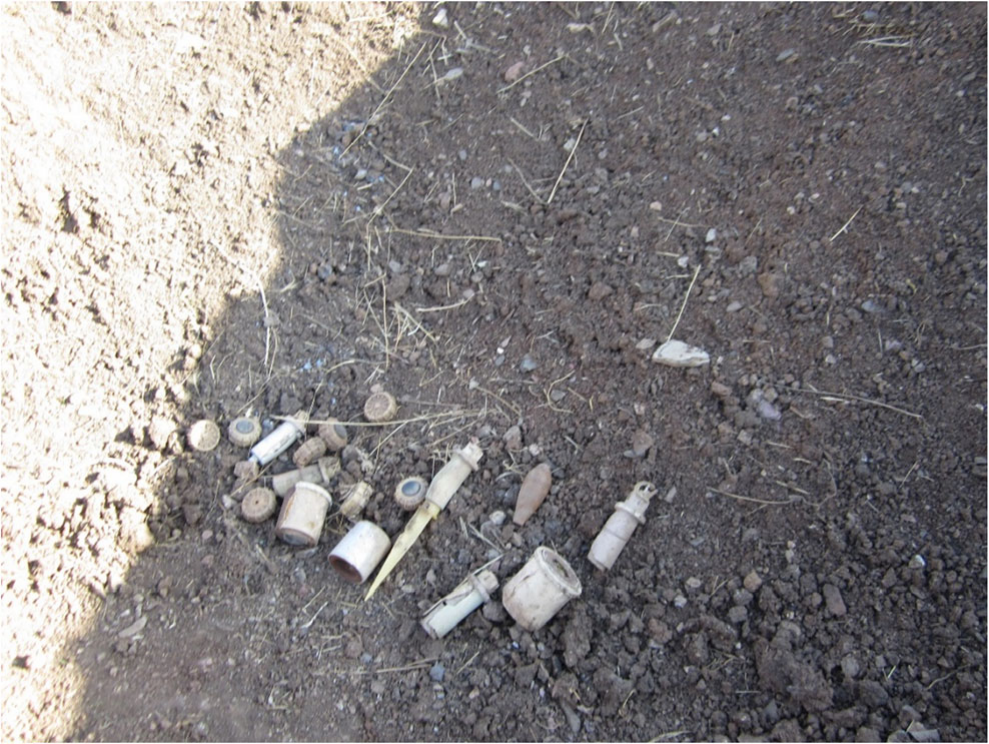
Anti-personal mines and unexploded remnant ordinance are collected and placed in the digging hole, which are ready to be detonated by the de-miner in the HSCZ (photo by R. Hamad 2016)

Figure 20 displays the typical finding mines that were placed in the digging hole and were ready to be detonated by de-miners at the HSNP site. The explosion is taking place inside a digging area in order to detonate the content of mines and other unexploded ordinance, followed by backfilling of the hole.

## Discussion

### Heavy metal contamination in soil in HSNP

The level of heavy metal contamination inside or around the holes or digging areas at two sites varied according to the quantity and type of mine explosions, geochemical characteristics of the soil and the rate of the heavy metals. Tables 2 and 3 show the heavy metal concentrations obtained from the test soil samples for sites 1 and 2. Some of the heavy metal concentrations decreased significantly with an increase in distance from the centres (source explosions) indicating high translocation in soil, while others were completely opposed such as Ni, Cr and Co only at site 2. The greater the mobility the higher the toxicity risk of these metals. Furthermore, in this study, underground soil was polluted by released heavy metal in HSNP. Thus, many dangerous heavy metals are likely to be deposited in the ground soil. These heavy metals can damage the normal activities and ecological balance of the underground soil.

In the present study, the level of soil accumulation of nickel and chromium occurred predominantly at centres at sites 1 and 2 (Figs. 4, 5, 6 and 7). Therefore, the concentration of Ni and Cr decreased significantly with the increase in distances from dugout pits at two sites. Similar to Ni and Cr, the concentration of cobalt also decreased with the increase in distances from dugout pits but only at site 2. The rest of heavy metals and cobalt at site 1 were totally opposed, which spatial distribution increased with the increase in distances. The concentration and the mobility of heavy metals in the current study show progressive variations, which may be ascribed to the variation in soil characteristics and the quantity of heavy metals that are released into the soil after the explosions of mines at two sides. The biggest problem comes when these contaminated lands are reused (used) as an agricultural land. This matter is certainly a concern in HSNP where productive agricultural land is concerned. Thus, the effects of soil pollution are massive on agricultural land and they can be reduced by soil fertility, increased erodibility, larger loss of soil and nutrients, reduced crop yield and imbalance of soil fauna and flora.

On the other hand, the variances of transferring different metals in soils might be explained by the following reasons or facts: the total concentration of metals has a big role in their distribution among the chemical fractions (Kashem et al. 2007). Another reason could be related between the mobility and bioavailability of these metals and their solubility and geochemical forms (Ma and Rao 1997). Another study examined the retention of lead, copper, nickel and zinc elements in soil with increasing pH from 7.0 to 7.5 (Harter 1983), whereas Kabata (2010) stated that there are “some of metals accumulated in soil are depleted slowly by leaching, plant uptake, erosion, or deflation”.

Globally, soil contamination induced by unexploded ordnance and remnants of war is still a big issue all over the world. For example, the contamination of soils still exists in France and Belgium over a century after the war. Hundreds of thousands of unexploded items of ordinance were deposited in the border area to be neutralised at the end of War World I. This resulted in the inhibition of tree growth in the contaminated areas where only a few lichens survive (https://www.riskope.com/2014/02/13/100-years-after-wwi-the-soil-between-france-and-belgium-is-still-contaminated-by-remnants-of-war-uxos-and-toxic-chemical-compound/). Another example is the large area of northern French agricultural land, which is heavily contaminated with harmful minerals as a result of recycling site for millions of First World War bombshells. Therefore, in the abovementioned area, sale of any agricultural products is prohibited until certain warranties are satisfied by the French government. Furthermore, throughout World War I, there were enough stockpiles of weapons and bombs that where lying around after achieving peace in 1918. To remove these hazards, many organisations appeared around that time and succeeded to destroy these bombs and weapons without any significant government interference. The organisations used many ways to get rid of these bombs and weapons, and many were detonated, burned, dismantled or drained into the soil. However, the French and Belgian governments are still facing the impact from World War I, where the Red Zone in France is still in place (https://m.warhistoryonline.com/war-articles/hitler-only-have-one.html). Soil contamination has also been documented on the former Soča front in Slovenia. Shrapnel and bullets that have remained in the ground before more than 90 years ago were the main reasons for elevated concentrations of copper, lead, zinc, mercury and tin in the soil. Accordingly, due to the large number of pollutants in abandoned disposal sites throughout Belgium, France and Germany, researchers have suggested that the surrounding land should not be used for agricultural purposes (http://www.toxicremnantsofwar.info/assessing-the-toxic-legacy-of-first-world-war-battlefields/).

To conclude, these types of studies are relevant for Halgurd-Sakran National Park as moving and distribution of heavy metals in soils are major concerns that affect the vegetables grown in contaminated soils and their possible consumption by animals or humans through food chain process.

### The contamination indices

The contamination of soils was assessed on the basis of enrichment factor, pollution load index and geo-accumulation index. The results show that variations of the same rate of heavy metal concentrations at two sites in the analysed soil confirmed an anthropogenic contribution due to the demining operation processes in the study areas. Thus, they are more strongly influenced by anthropogenic activities than naturally occurring sources as reflected by a high degree of contamination of toxic elements released from the content of mines and other remnant ordinance that are released into the soils.

The results of the geochemical analysis show that the high accumulation of Ni and Cr at two sites and Co at site 2 can be a serious concern. Furthermore, they appear to have a stronger contribution in the heavy metals released into the soil following the mines and unexploded ordinance explosions. The variations in concentrations of heavy metals are quantity and type dependent of mines and other unexploded ordinance.

Distances of sampling locations were measured from a location in the centre of the hole that the explosions took place at two sites in Halgurd-Sakran National Park. The evaluation of the current state of the environmental quality of soils of two different polluted areas at the same time enables a comparison of contamination indexes. This information in such areas as national parks can be useful in guiding planners from the view of soil protection and providing more reasonable results of transferring heavy metals in soils. Furthermore, calculating the different contamination indexes based on distance from the source of pollution in different areas reflects the properties and characterisation of different metals through their transportation in soils.

**Fig. 21:**
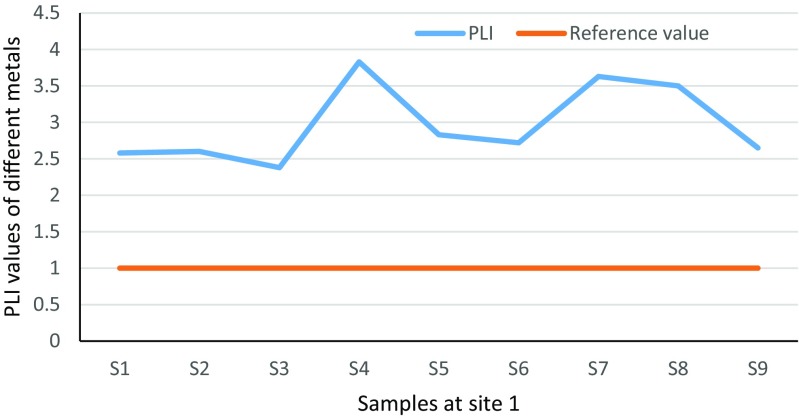
Site 1 pollution load index values of Ni, Cr, Co, Mn, Zn, Cu, Pb and As that exceeded the reference value of 1 in HSNP

**Fig. 22:**
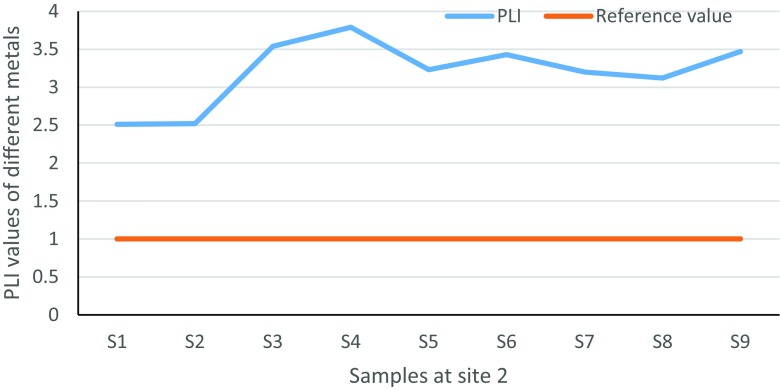
Site 2 pollution load index values of Ni, Cr, Co, Mn, Zn, Cu, Pb and As that exceeded the reference value of 1 in HSNP

Lastly, there are some tools that quickly assess the presence and intensity of anthropogenic contaminant deposition on surface soil such as contamination factor and pollution load index. PLI is a quick tool to determine the status of the contamination in different locations. Figures 21 and 22 illustrate that all sampling locations at two sites have exceeded the value of 1, which indicates the total pollution in the areas.

## Conclusions

An assessment of heavy metal contamination by Ni, Cr, Mn, Co, Cu, Zn, As and Pb was carried out on the soils of two sites in Halgurd-Sakran based on EF, PLI and I-geo. The distribution pattern of trace metals in the soils of the two sites indicates high pollution. Ni and Cr with Co at site 2 were the main polluting heavy metals with extremely high values. The high values of Ni and Cr in samples 4 and 3 from site 1 and site 2, respectively, are related to the operation of the explosion at these two points. The aforementioned (abovementioned) connection can also include Co at site 2. Therefore, these two sites present several potential hazards that need to be remediated by soil amendments. However, I-geo and PLI values indicated widespread pollution by Ni, Cr and Mn in the soils.

The application of aforementioned indices indicated anthropogenic contribution mainly by the elements Ni and Cr, which certainly originated from the explosions at two sites in HSNP. Therefore, due to the toxicity of heavy metals, especially at the explosion places, the use of these areas for agricultural purposes should be discouraged as plants and vegetables can easily absorb them at elevated levels, which contain many heavy metals. Through time, the whole area may contaminate with the toxic elements. Nickel was among the elements that have highest rate in soil, which may cause significant environmental pollution. The greatest Ni and Co concentrations obtained from the geochemical samples were found to be in the centre at two sites. Thus, high concentrations of Ni and Co may present potential health risk for the human populations residing in the surrounding area of the whole area. Furthermore, metal concentrations of Cr, Zn, Mn, As and Cu had the highest rate at an average distance of 10 m from the contaminating source at site 1, whereas metal concentration of Zn, Mn, As, Cu and Pb had the highest rate at a distance of 10 m from the contaminating source at site 2. Thus, the highest contamination occurs within a 10-m circle from the contaminating source.

By continuous application of heavy metals that are released into the soil after mine clearing operation, the metals are accumulated into the soils and plants. Therefore, it requires some management strategies and steady observation is recommended to prevent future health problems. Soil and geological investigations are necessary at the places that explosions are conducted in order to trace the metals.

Finally, the results indicate that the heavy metals are present in the soil as part of pollution loads produced by anthropogenic inputs at two different sites in HSNP. Their entrance into the food is a geochemical hazard because of their toxicity to human health.
